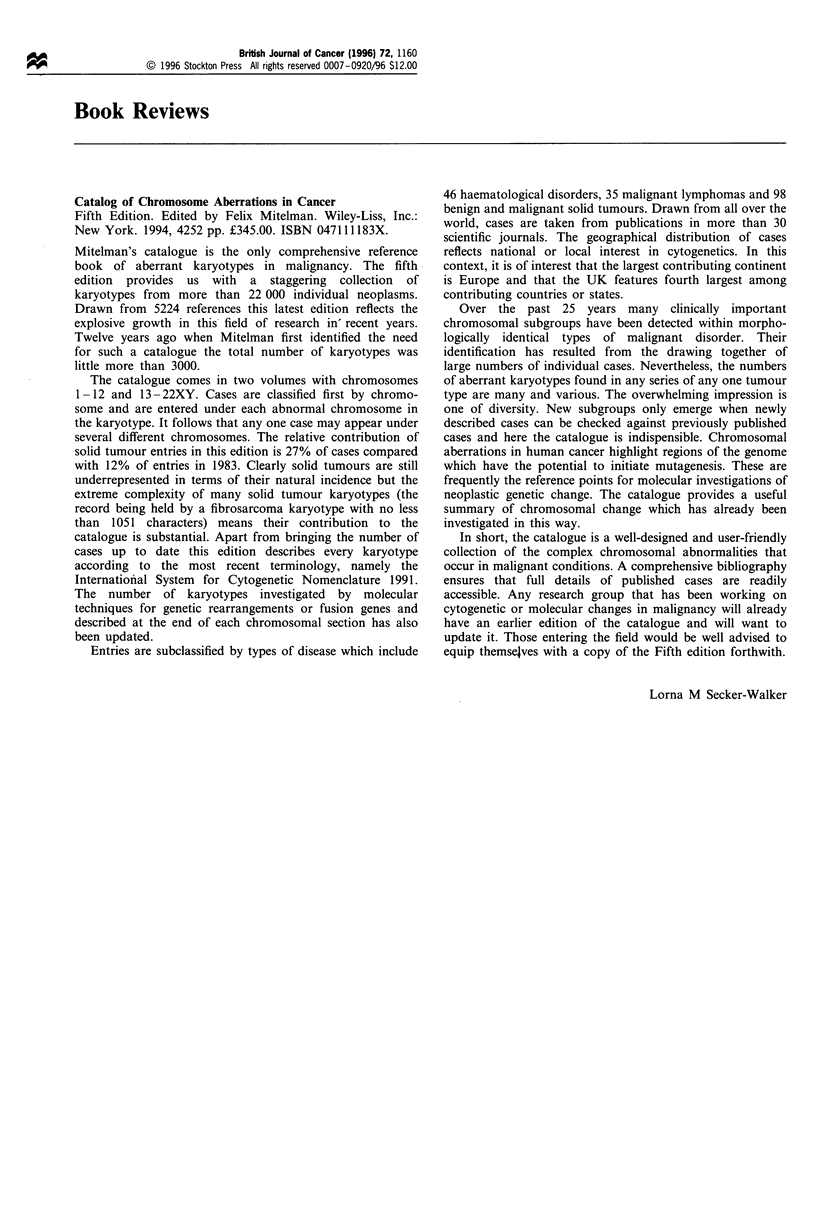# Catalog of Chromosome Aberrations in Cancer

**Published:** 1996-05

**Authors:** Lorna M Secker-Walker


British Journal of Cancer (1996) 72, 1160
© 1996 Stockton Press All rights reserved 0007-0920/96 $12.00

### Book Reviews

**Catalog of Chromosome Aberrations in Cancer**
Fifth Edition. Edited by Felix Mitelman. Wiley-Liss, Inc.: New York. 1994, 4252 pp. £345.00. ISBN 047111183X.

Mitelman's catalogue is the only comprehensive reference book of aberrant karyotypes in malignancy. The fifth edition provides us with a staggering collection of karyotypes from more than 22 000 individual neoplasms. Drawn from 5224 references this latest edition reflects the explosive growth in this field of research in recent years. Twelve years ago when Mitelman first identified the need for such a catalogue the total number of karyotypes was little more than 3000.

The catalogue comes in two volumes with chromosomes 1–12 and 13–22XY. Cases are classified first by chromosome and are entered under each abnormal chromosome in the karyotype. It follows that any one case may appear under several different chromosomes. The relative contribution of solid tumour entries in this edition is 27% of cases compared with 12% of entries in 1983. Clearly solid tumours are still underrepresented in terms of their natural incidence but the extreme complexity of many solid tumour karyotypes (the record being held by a fibrosarcoma karyotype with no less than 1051 characters) means their contribution to the catalogue is substantial. Apart from bringing the number of cases up to date this edition describes every karyotype according to the most recent terminology, namely the International System for Cytogenetic Nomenclature 1991. The number of karyotypes investigated by molecular techniques for genetic rearrangements or fusion genes and described at the end of each chromosomal section has also been updated.

Entries are subclassified by types of disease which include 46 haematological disorders, 35 malignant lymphomas and 98 benign and malignant solid tumours. Drawn from all over the world, cases are taken from publications in more than 30 scientific journals. The geographical distribution of cases reflects national or local interest in cytogenetics. In this context, it is of interest that the largest contributing continent is Europe and that the UK features fourth largest among contributing countries or states.

Over the past 25 years many clinically important chromosomal subgroups have been detected within morphologically identical types of malignant disorder. Their identification has resulted from the drawing together of large numbers of individual cases. Nevertheless, the numbers of aberrant karyotypes found in any series of any one tumour type are many and various. The overwhelming impression is one of diversity. New subgroups only emerge when newly described cases can be checked against previously published cases and here the catalogue is indispensible. Chromosomal aberrations in human cancer highlight regions of the genome which have the potential to initiate mutagenesis. These are frequently the reference points for molecular investigations of neoplastic genetic change. The catalogue provides a useful summary of chromosomal change which has already been investigated in this way.

In short, the catalogue is a well-designed and user-friendly collection of the complex chromosomal abnormalities that occur in malignant conditions. A comprehensive bibliography ensures that full details of published cases are readily accessible. Any research group that has been working on cytogenetic or molecular changes in malignancy will already have an earlier edition of the catalogue and will want to update it. Those entering the field would be well advised to equip themselves with a copy of the Fifth edition forthwith.

Lorna M Secker-Walker